# Protective Effect of Ethanolic Extract of *Tabernaemontana divaricata* (L.) R. Br. against DEN and Fe NTA Induced Liver Necrosis in Wistar Albino Rats

**DOI:** 10.1155/2014/240243

**Published:** 2014-07-17

**Authors:** Kannappan Poornima, Palanisamy Chella Perumal, Velliyur Kanniappan Gopalakrishnan

**Affiliations:** ^1^Department of Biochemistry, Karpagam University, Coimbatore, Tamil Nadu 641021, India; ^2^Department of Bioinformatics, Karpagam University, Coimbatore, Tamil Nadu 641021, India

## Abstract

This study is an attempt to evaluate the hepatoprotective activity of* Tabernaemontana divaricata* against DEN and Fe NTA induced liver necrosis in rats. Ethanolic extract of the whole plant of* Tabernaemontana divaricata* at doses of 200 and 400 mg/kg body weight and 5-fluorouracil (standard drug) was orally administered to male Wistar Albino rats once daily for 24 weeks, simultaneously treated with the carcinogen DEN and Fe NTA. In simultaneously treated animals, the plant extract significantly decreased the levels of uric acid, bilirubin, AST, ALT, and ALP in serum and increased the levels of liver marker enzymes in liver. Treatment with the extracts resulted in a significant increase in the levels of antioxidants accompanied by a marked reduction in the levels of malondialdehyde when compared to DEN and Fe NTA treated group. When compared with 200 mg/kg bw rats, 400 mg/kg bw rats and 5-fluorouracil treated rats showed better results in all the parameters. The histopathological studies confirmed the protective effects of extract against DEN and Fe NTA induced liver necrosis. Thus, it could be concluded that the use of* Tabernaemontana divaricata* extract in the treatment of carcinogen induced hepatic necrosis.

## 1. Introduction

Liver is the most important organ, which plays a pivotal role in regulating various physiological processes in the body [1]. It is involved in several vital functions, such as metabolism, secretion, and storage. It has great capacity to detoxicate toxic substances and synthesize useful principles. Therefore, damage to the liver inflicted by hepatotoxic agents is of grave consequences [2].

Oxidative stress, an important factor that induces liver fibrosis, represents a key feature of hepatitis induced by various conditions, including anoxic/reoxygenation injury, autoimmune hepatitis, viral hepatitis, and alcoholic hepatitis [3]. Less severe oxidative stress may sustain fibrosis progression by the activation and morphological change in hepatic stellate cells (HSCs) that include promoting proliferative activity, synthesis, and degradation/remodeling of the extracellular matrix, chemotaxis, contractility, proinflammatory activity, and retinoid loss [4]. Ferric nitrilotriacetic acid (Fe-NTA) and diethyl nitrosamine (DEN) are used extensively to induce oxidative stress and assumed to initiate free radical mediated lipid peroxidation leading to the accumulation of lipid-derived oxidation products that cause liver injury and excess collagen deposition in the liver, resulting in liver necrosis [5]. Conventional or synthetic drugs used in the treatment of liver diseases are sometimes inadequate and can have serious adverse effects [6]. On the other hand, Ayurveda, an indigenous system of medicine in India, has a long tradition of treating liver disorders with plant drugs [7].

*Tabernaemontana divaricata* (L.) R. Br. is a glabrous, evergreen, dichotomously branched shrub, belonging to the family Apocynaceae. It is a shrub very common in India and grows to a height of 6–8 feet. It is distributed in upper Ganjetic plain, Garhwal, East Bengal, Assam, Karnataka, Kerala, Tamilnadu, and Burma [8]. It has been extensively investigated and rich in a number of chemical constituents such as alkaloids, triterpenoids, steroids, flavanoids, phenylpropanoids, and phenolic acids. In Ayurveda, the root is acrid; bitter with a flavor; digestible; useful in Kapha, biliousness, and diseases of the blood [9]. It is aphrodisiac; tonic especially to the brain, liver, and spleen and it is also a purgative. The milky juice mixed with oil is rubbed on to the head to cure the pain in the eye. The milky juice is very useful in many eye infections, especially red eye. It kills intestinal worms, and its root when chewed relieves tooth ache. Herbal medicines have been proved to be powerful therapeutics for treatment of various human sufferings including cancer, arteriosclerosis, ulcer, diabetes, kidney diseases, and liver diseases [10]. The ethanol and aqueous extracts of* Tabernaemontana divaricata* (L.) R. Br. flower possess very good in vitro superoxide, hydroxyl and nitric oxide radical scavenging, and lipid peroxidation inhibiting activities [8, 11] The phytochemical screening of the plant extract showed the presence of alkaloids, saponin, tannin, phenolic compounds, flavonoids, cardioglycosides, terpenoid, amino acids, proteins, and carbohydrates [8]. The toxicological evaluation study was already carried out. The plant extract is safe up to 2000 mg/kg body weight. The subacute study also showed no abnormalities in biochemical and marker enzymes of liver and kidney [12]. On the basis of folklore usage and previous research findings, a systematic research was undertaken to evaluate the protective effect of ethanolic extract of* Tabernaemontana divaricata* (L.) R. Br. against DEN and Fe NTA induced liver necrosis in male Wistar albino rats by in vivo model.

## 2. Materials and Methods

### 2.1. Plant Collection and Authentication

The whole plant of* Tabernaemontana divaricata* (L.) R. Br. was collected in and around Coimbatore district of Tamil Nadu, India, and authenticated by Dr. P. Satyanarayana, Scientist D, Botanical Survey of India, Coimbatore, Tamil Nadu, India. The Voucher number is BSI/SRC/5/23/2011-12/Tech. 1538. The whole plant was cut into pieces, cleaned, air dried at 25°C for 10 days in the absence of sunlight, and powdered well into a coarse powder using a mixer. Then they were weighed and kept in an airtight container and stored in refrigerator for future use.

### 2.2. Preparation of Extract

50 g of the powdered whole plant material of* Tabernaemontana divaricata* (L.) R. Br. was extracted by continuous extraction using soxhlet apparatus with the solvent ethanol for 72 hrs. The resultant extract was concentrated to dryness under reduced pressure below 40°C in rotary evaporator and stored at 4°C in a tightly capped brown bottle and kept in refrigerator for the study. According to OECD guidelines, the doses were determined and administered.

### 2.3. Preparation of Fe NTA Solution

Fe NTA solution was prepared fresh immediately before its use by the method of Awai et al. [13] as modified by Athar and Iqbal [14]. To prepare Fe NTA, ferric nitrate (0.16 mmol/kg body weight) solution was mixed with fourfold molar excess of disodium salt of NTA (0.64 nmol) and the pH was adjusted to 7.4 with sodium bicarbonate solution. The concentration of the Fe NTA solution prepared was 10 mL/kg body weight and the dose was 9 mg Fe/kg body weight.

### 2.4. Experimental Design

#### 2.4.1. Animals

Adult male Albino rats weighing about 120–140 g were obtained from the animal house of Karpagam Arts and Science College, Coimbatore, India. The animals were maintained under standard conditions and were housed four per cage in polypropylene cages with a wire mesh top and a hygienic bed of husk in a specific pathogen free animal room under controlled conditions of 12 h light and 12 h dark cycle, with temperature of 24 ± 2°C and relative humidity of 50 ± 10%, and fed with rodent diet and water* ad libitum.* The study was approved by the Committee for the Purpose of Control and Supervision of Experiments on Animals (CPCSEA), Government of India.

#### 2.4.2. Induction of Carcinogen

The carcinogenesis was initiated by a single intraperitoneal injection of DEN (diethyl nitrosamine) at a dose level of 200 mg/kg body weight. After ten days the carcinogenesis is promoted by Fe-NTA at a dose level of 9 mg Fe/kg body weight (ip) twice a week for 24 weeks.

#### 2.4.3. Experimental Protocol

Forty-two albino rats were randomly allocated to seven groups of six rats each.

 Group I received only normal food and water.
 Group II received a single intraperitoneal injection of DEN at a dose level of 200 mg/kg body weight in the first day and after ten days the carcinogenesis was promoted by Fe NTA at a dose level of 9 mg Fe/kg body weight (ip) twice a week for twenty-four weeks.
 Groups III and IV were induced with carcinogen and simultaneously treated with the plant extract (200 and 400 mg/kg bw), respectively, daily for twenty-four weeks.
 Groups V and VI received only the plant extract (200 and 400 mg/kg bw), respectively, daily for twenty four weeks.
 Group VII animals were induced with carcinogen and simultaneously treated with the standard drug, 5 fluorouracil (20 mg/kg bw), for twenty-four weeks.

All rats were sacrificed 12 h after the experimental period. Blood was collected and the serum was separated.

#### 2.4.4. Preparation of Tissue Homogenate

Liver was removed quickly and washed in ice cold isotonic saline. The tissue was homogenized in 0.1 M Tris-HCl buffer of pH 7.4 at 4°C in a potter homogenizer, at 600 rpm for 3 minutes. The filtrate was used for further estimations.

#### 2.4.5. Estimation of Biochemical Parameters

Aliquot of the diluted serum was used for the estimation of serum biomarkers like uric acid [15], bilirubin [16], aspartate transaminase (AST) [17], alanine transaminase (ALT) [17], and alkaline phosphatase (ALP) [18] with their respective methods.

#### 2.4.6. Estimation of Liver Marker Enzymes in Liver

Estimation of liver marker enzymes such as aspartate transaminase (AST) [17], alanine transaminase (ALT) [17], alkaline phosphatase (ALP) [18], acid phosphatase (ACP) [19], lactate dehydrogenase (LDH) [20], and 5′ nucleotidase [21] was estimated in the liver tissue homogenate by the respective methods.

#### 2.4.7. Estimation of Lipid Peroxidation

Lipid peroxidation was measured by the thiobarbituric acid (TBA) reaction method [22]. In brief, samples were mixed with TBA reagent consisting of 0.375% TBA and 15% trichloroacetic acid in 0.25-N hydrochloric acid. The reaction mixtures were placed in a boiling water bath for 30 min and centrifuged at 1800 g for 5 min. The absorbance of the supernatant was measured at 535 nm. MDA, a measure of lipid peroxidation, was calculated using an extinction coefficient of 1.56 × 105/M cm. The results were expressed as *μ*M/mg protein.

#### 2.4.8. Antioxidant Assays

The liver homogenate was used to analyze the enzymatic antioxidant activities by superoxide dismutase (SOD) [23], catalase (CAT) [24], glutathione peroxidase (GPx) [25], glutathione-S-transferase (GST), peroxidase (Px) [26], and glucose-6-phosphate dehydrogenase (G6PD) [27], and nonenzymatic antioxidants like reduced glutathione (GSH) [28], total sulphydryl group [29], and vitamin C [30] were evaluated in liver tissue homogenate by respective methods.

#### 2.4.9. Histopathological Studies

For histological studies, the liver tissues were fixed with 10% phosphate buffered neutral formalin, dehydrated in graded (50%–100%) alcohol and embedded in paraffin. Thin sections (5 *μ*m) were cut and stained with routine hematoxylin and eosin (H&E) stain for photo microscopic assessment. The initial examination was qualitative, with the purpose of determining histopathological lesions in liver.

### 2.5. Statistical Analysis

Results were expressed as mean ± SD. The statistical comparison among the groups was performed with one-way ANOVA test using a statistical package program (SPSS 10.0) at *P* < 0.05 significant level.

## 3. Results and Discussion

### 3.1. Estimation of Biochemical Parameters

Table 1 shows the level of uric acid, bilirubin, AST, ALT, and ALP in serum. The levels of uric acid, bilirubin, AST, ALT, and ALP were increased in DEN initiated and Fe NTA promoted group II animals when compared to the control (group I) animals. Increased levels of uric acid and bilirubin were significantly decreased in group III rats treated with 200 mg/kg bw of the ethanolic extract of* Tabernaemontana divaricata *(L.) R. Br. whereas in groups IV and VII rats treated with 400 mg/kg bw of the plant extract and the standard drug, respectively, the levels were normalized as those of control. Group V and group VI rats were compared with control group I and they showed no significant change.

### 3.2. Estimation of Liver Marker Enzymes in Liver

The liver marker enzymes like AST, ALT, ACP, ALP, LDH, and 5′ nucleotidase of both control and experimental groups were shown in Table 2. A significant (*P* < 0.05) reduction in the activity of the enzymes AST, ALT, ACP, ALP, LDH, and 5′ nucleotidase in group II of experimental animals was noticed. These marker enzymes were significantly increased (*P* < 0.05) in groups III, IV, and VII animals (plant extract and standard drug treated rats). No significant difference has been observed in the plant extract alone treated groups (groups V and VI) and the control group.

### 3.3. Lipid Peroxidation and Antioxidant Assays

#### 3.3.1. Lipid Peroxidation and Enzymatic Antioxidant Assays

The changes in the lipid peroxidation and enzymatic antioxidants in the liver are illustrated in Table 3. From the table it is found that the level of lipid peroxidation is increased (*P* < 0.05) significantly and a significant reduction (*P* < 0.05) in the activity of antioxidant enzymes were noted in rats intoxicated with DEN and Fe NTA (group II) when compared to control (group I). The decline of antioxidant status partially explains the mechanism of hepatotoxicity and enhanced radical production by DEN and Fe NTA, whereas in groups III, IV and VII rats treated with 200 mg and 400 mg/kg bw of ethanolic extract of* Tabernaemontana divaricata* (L.) R. Br. and the standard drug 5 fluorouracil, respectively, the level of these parameters was significantly reverted back to near normal level when compared to group II animals. This shows the ability of the plant extract to scavenge the free radicals by enhancing the enzymatic antioxidants thereby reducing the oxidative stress.

#### 3.3.2. Nonenzymatic Antioxidants

Table 4 exhibits the levels of nonenzymatic antioxidants in liver of control and experimental animals.

From the table it is found that the level of GSH, TSH, and vitamin C was significantly reverted back to near normal level when treated with the plant extracts (200 and 400 mg/kg bw) and the standard drug when compared to group II treated with DEN and Fe NTA.

### 3.4. Histopathological Studies

**Figure 1:**
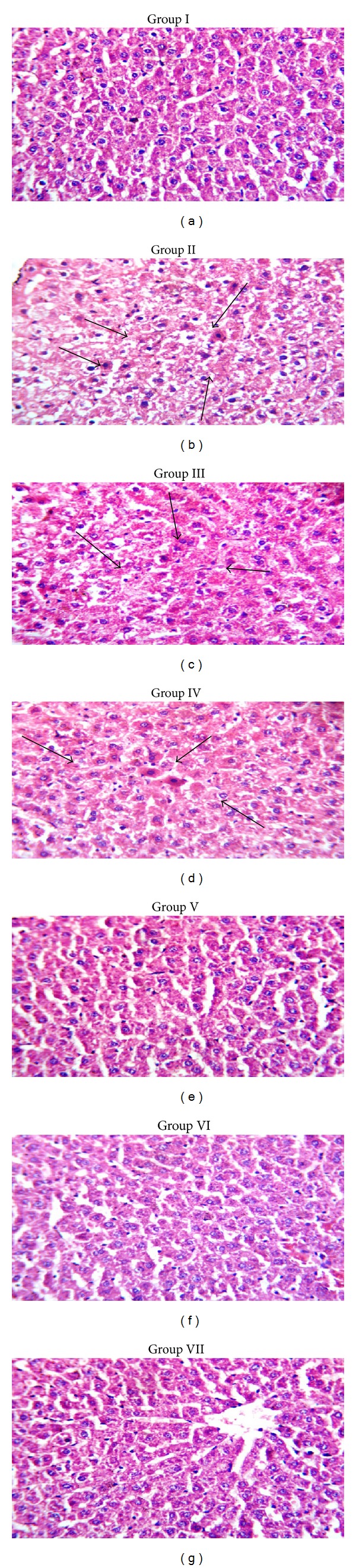
Histopathology of liver in control and experimental rats. (a) Normal control. (b) DEN and Fe NTA induced. (c) DEN and Fe NTA +* Tabernaemontana divaricata* (L.) R. Br. (200 mg/kg body weight). (d) DEN and Fe NTA +* Tabernaemontana divaricata* (L.) R. Br. (400 mg/kg body weight). (e)* Tabernaemontana divaricata* (L.) R. Br. (200 mg/kg body weight). (f)* Tabernaemontana divaricata* (L.) R. Br. (400 mg/kg body weight). (g) DEN and Fe NTA + standard drug (5 fluorouracil).

Figure 1 shows the histopathology of liver in both control and experimental groups. Group I shows the normal structure of liver. The portal tracts, central veins, hepatocytes of all zones (zones 1, 2, and 3), sinusoids, and Kuppfer cells appear normal. In group II rats treated with DEN and Fe NTA for a period of 24 weeks, the sections studied show that there is patchy hepatocellular necrosis. The portal tract and central veins appeared normal. There is no obvious dysplasia or malignancy. In group III the experimental animals showed a focal mild hepatocellular necrosis in some parts and the rest of the liver tissue is normal. The portal tracts, central veins, and sinusoids appeared normal. This shows that the treatment has some effect and it is moving to the recovery phase and it was confirmed with group IV rats, in which the rats were treated with carcinogen and the plant extract 400 mg/kg body weight showed no evidence of hepatocellular necrosis. The portal tracts, central veins, and sinusoids appeared normal; this shows the effective nature of the extract. In group V and group VI no abnormal changes were seen and the results are similar to those of the control. This shows the safety nature of the plant extract. In standard drug treated group (group VII) the results are similar to those of control. This confirms the safety and effective nature of the ethanolic extract of* Tabernaemontana divaricata* (L.) R. Br. against DEN and Fe NTA.

## 4. Discussion

Liver plays a pivotal role in regulation of physiological processes such as metabolism, secretion, and storage. Unfortunately it is a common target for a number of toxicants [31]. An iron chelate, ferric nitrilotriacetate (Fe-NTA), induces necrosis as a consequence of lipid peroxidation and oxidative tissue damage that eventually leads to high incidence of cancer in liver and kidney. Fe-NTA acts through the generation of free radicals and by enhancing the rate of DNA synthesis with simultaneous decrease in antioxidant defenses.

However, unfortunately, the modern system of medicine offers only limited options for its cure. Thus, there is a great necessity for developing alternative strategies to cope with it. Use of safe plant based compounds is one of the most valuable options to prevent and/or delay the neoplastic inception. Consequently, a lot of scientific research is currently focused on exploration of safe and effective phytochemicals for the management of liver necrosis. Plants produce a large number of naturally occurring secondary metabolites, many of which have unique pharmacologic activities [32, 33].

In our present study the ethanolic extract of* Tabernaemontana divaricata* (L.) R. Br. showed a good hepatoprotective activity against DEN and Fe NTA induced liver necrosis. The increased levels of marker enzyme in serum and decreased level in the liver are indicators of cellular damage and loss of functional integrity of the cell membrane due to DEN and Fe NTA administration. Treatment with the ethanolic extract of* Tabernaemontana divaricata* (L.) R. Br. brought back these enzymes to near normal level by probably preserving the functional integrity of the hepatocytes showing its hepatoprotective action against DEN and Fe NTA induced liver damage. The present study was in accordance with that of Langaswaran et al. [34].

The ethanolic extract of* Tabernaemontana divaricata* (L.) R. Br. showed that the increased activity of GST supports the detoxification capacity and the degradation of electrophilic metabolites by this enzyme. Sangeetha and Krishnakumari [35] reported that the decreased activity of hepatic markers in carbon tetrachloride induced toxicity was increased by treatment with* Tephrosia purpurea*. Gupta et al. [36] reported that the MEBR extract treated animals restored the hepatic lipid peroxidation and free radical scavenging enzyme GSH as well as antioxidant enzymes such as SOD and CAT in tumour bearing mice to near normal levels. In the present study, DEN and Fe NTA induced fall of AST and ALT in liver associated with rise in plasma suggests the extent of liver damage and release of these enzymes from the damaged liver cells and disruption of cellular integrity. The decrease in the liver AST and ALT supports the hypothesis of hepatocellular necrosis. It is also confirmed by histopathological examinations that the abnormalities produced by the carcinogen is reverted back to near normal in plant extract (200 and 400 mg/kg bw) treated groups of rats.

## 5. Conclusion

To conclude, the plant has a very good antioxidant and hepatoprotective effect against liver cell necrosis induced by DEN and Fe-NTA in male Wistar Albino rats and it can be used for the purpose of hepatoprotection.

## Figures and Tables

**Table 1:** Changes in the level of uric acid, bilirubin, AST, ALT, and ALP in serum.

Particulars	Group I	Group II	Group III	Group IV	Group V	Group VI	Group VII
Uric acid (mg/dL)	1.54 ± 0.01^a^	3.24 ± 0.02^b^	2.2 ± 0.01^c^	1.56 ± 0.01^a^	1.5 ± 0.03^d^	1.39 ± 0.05^e^	1.42 ± 0.08^e^
Bilirubin (mg/dL)	0.9 ± 0.05^a^	2.37 ± 0.06^b^	1.31 ± 0.06^c^	0.86 ± 0.07^a^	0.9 ± 0.05^a^	0.87 ± 0.03^a^	0.85 ± 0.05^a^
AST (IU/L)	32.85 ± 1.59^a^	101.13 ± 0.86^b^	57.32 ± 1.51^c^	34.39 ± 0.86^d^	32.85 ± 1.12^a^	32.68 ± 0.77^a^	34.03 ± 0.92^ad^
ALT (IU/L)	68.99 ± 0.49^a^	138.77 ± 2.97^b^	90.59 ± 0.79^c^	69.85 ± 0.24^a^	72.89 ± 1.31^d^	68.69 ± 0.71^a^	73.24 ± 0.48^d^
ALP (IU/L)	72.94 ± 0.32^a^	254.0 ± 0.45^b^	141.56 ± 0.41^c^	73.65 ± 0.38^d^	72.62 ± 0.41^a^	70.68 ± 0.64^a^	74.1 ± 0.63^d^

Values are expressed as mean ± SD (*n* = 6).

Values not sharing the common superscript differ significantly (DMRT).

**Table 2:** Changes in the activities of liver marker enzymes in liver.

Particulars	Group I	Group II	Group III	Group IV	Group V	Group VI	Group VII
AST	77.94 ± 2.48^a^	44.9 ± 1.71^b^	68.47 ± 1.79^c^	72.66 ± 1.77^d^	76.46 ± 1.17^a^	76.71 ± 1.3^a^	75.85 ± 1.1^a^
ALT	36.25 ± 0.37^a^	26.94 ± 0.71^b^	30.17 ± 0.72^c^	35.37 ± 0.94^a^	36.14 ± 0.46^a^	34.82 ± 0.46^a^	33.73 ± 0.73^d^
ALP	12.95 ± 0.49^a^	7.86 ± 0.66^b^	9.93 ± 0.39^c^	12.85 ± 0.34^a^	12.0 ± 0.4^a^	12.32 ± 0.34^a^	12.32 ± 0.57^a^
ACP	9.39 ± 0.1^a^	4.67 ± 0.15^b^	7.51 ± 0.12^c^	9.02 ± 0.07^d^	9.45 ± 0.16^a^	9.32 ± 0.23^a^	9.27 ± 0.15^a^
LDH	86.51 ± 0.76^a^	52.33 ± 0.64^b^	66.28 ± 0.65^c^	83.71 ± 0.94^d^	84.98 ± 0.78^e^	85.58 ± 0.5^e^	85.36 ± 0.88^a^
5′ nucleotidase	8.63 ± 0.04^a^	5.09 ± 0.05^b^	6.4 ± 0.06^c^	8.14 ± 0.07^d^	8.44 ± 0.17^a^	8.53 ± 0.05^a^	8.31 ± 0.06^d^

Values are expressed as mean ± SD (*n* = 6).

Values not sharing the common superscript differ significantly (DMRT).

Units are as follows:

AST, ALT, LDH—nmoles of pyruvate liberated/min/mg protein.

ALP, ACP—nmoles of phenol liberated/min/mg protein.

5′ nucleotidase—nmoles of phosphate liberated/min/mg protein.

**Table 3:** Changes in the level of lipid peroxidation and enzymatic antioxidants in liver.

Particulars	Control (Group I)	DEN + Fe-NTA (Group II)	DEN & Fe-NTA + plant extract (200 mg/kg) (Group III)	DEN & Fe-NTA + plant extract (400 mg/kg) (Group IV)	Plant extract alone (200 mg/kg) (Group V)	Plant extract alone (400 mg/kg) (Group VI)	DEN & Fe-NTA + Std drug (GroupVII)
Lipid peroxidation in liver	1.56 ± 0.06^a^	4.95 ± 0.14^b^	2.56 ± 0.08^c^	1.74 ± 0.09^d^	1.58 ± 0.06^a^	1.54 ± 0.06^a^	1.59 ± 0.06^a^
SOD	11.08 ± 0.46^a^	6.19 ± 0.30^b^	9.96 ± 0.23^c^	10.92 ± 0.54^a^	11.39 ± 0.22^a^	12.62 ± 0.28^a^	10.41 ± 0.42^a^
Catalase	6.01 ± 0.05^a^	3.25 ± 0.26^b^	4.33 ± 0.08^c^	6.04 ± 0.09^a^	5.96 ± 0.07^a^	6.19 ± 0.07^a^	6.03 ± 0.03^a^
PX	40.22 ± 0.59^a^	16.85 ± 0.91^b^	35.64 ± 0.16^c^	39.33 ± 0.65^d^	39.30 ± 0.38^d^	38.94 ± 0.4^d^	39.19 ± 0.34^d^
GST	131.22 ± 2.7^a^	98.08 ± 2.84^b^	112.86 ± 2.62^c^	130.07 ± 0.60^a^	130.66 ± 1.27^a^	132.56 ± 0.65^a^	131.08 ± 1.99^a^
G6PD	13.95 ± 0.11^a^	5.46 ± 0.24^b^	8.67 ± 0.09^c^	13.51 ± 0.4^a^	13.8 ± 0.21^a^	13.88 ± 0.12^a^	13.7 ± 0.18^a^
GPx	43.94 ± 0.34^a^	21.58 ± 0.59^b^	30.64 ± 0.24^c^	45.06 ± 0.59^d^	45.54 ± 0.44^d^	46.59 ± 0.39^e^	46.16 ± 0.7^e^

Values are expressed as mean ± SD (*n* = 6).

Values not sharing the common superscript differ significantly (DMRT).

Units are as follows: SOD—unit/mg protein; CAT—*µ* moles of H_2_O_2_ consumed/min/mg protein;

Px—units/g tissue:

LPO—nmoles of MDA formed/mg protein.

**Table 4:** Changes in the level of nonenzymatic antioxidants in liver.

Particulars	Control (Group I)	DEN + Fe-NTA (Group II)	DEN & Fe-NTA + plant extract (200 mg/kg) (Group III)	DEN & Fe-NTA + plant extract (400 mg/kg) (Group IV)	Plant extract alone (200 mg/kg) (Group V)	Plant extract alone (400 mg/kg) (Group VI)	DEN & Fe-NTA + Std drug (GroupVII)
GSH	21.28 ± 0.45^a^	11.76 ± 0.59^b^	16.26 ± 0.65^c^	19.81 ± 0.14^d^	20.74 ± 0.42^a^	21.34 ± 0.32^a^	20.02 ± 0.34^d^
TSH	6.7 ± 0.07^a^	3.5 ± 0.05^b^	4.46 ± 0.06^c^	6.68 ± 0.06^a^	6.5 ± 0.04^a^	6.56 ± 0.08^a^	6.57 ± 0.08^a^
Vitamin C	3.0 ± 0.1^a^	1.82 ± 0.18^b^	2.8 ± 0.11^c^	3.02 ± 0.12^a^	3.09 ± 0.05^a^	3.13 ± 0.12^a^	2.84 ± 0.11^d^

Values are expressed as mean ± SD (*n* = 6).

Values not sharing the common superscript differ significantly (DMRT).

The units were expressed in *µ*g/mg protein.
